# Association of physician-delivered virtual care near the end of life with healthcare use outcomes: A national population-based study of Canadians

**DOI:** 10.1371/journal.pone.0324898

**Published:** 2025-06-03

**Authors:** Mohammed Rashidul Anwar, Rabia Akhter, Thérèse A. Stukel, Hannah Chung, Chaim M. Bell, James Downar, Nathan Stall, Peter Tanuseputro, Aynharan Sinnarajah, Sandra Peterson, Asmita Bhattarai, John C. Knight, Kieran L. Quinn

**Affiliations:** 1 Institute of Health Policy, Management and Evaluation, University of Toronto, Toronto, ON, Canada; 2 Temmy Latner Centre for Palliative Care, Sinai Health System, Toronto, ON, Canada; 3 ICES, Toronto and Ottawa, ON, Canada; 4 Department of Medicine, University of Toronto, Toronto, ON, Canada; 5 Department of Medicine, Sinai Health System, Toronto, ON, Canada; 6 Division of Palliative Care, Department of Medicine, University of Ottawa, Ottawa, ON, Canada; 7 Bruyere Research Institute, Ottawa, ON, Canada; 8 Queensland University of Technology School of Law, Brisbane City, Queensland, Australia; 9 Department of Family Medicine and Primary Care, Li Ka Shing Faculty of Medicine, The University of Hong Kong, Hong Kong SAR, China; 10 Department of Medicine, Queen’s University, Kingston, ON, Canada; 11 Centre for Health Services and Policy Research, The University of British Columbia, Vancouver, BC, Canada; 12 Department of Community Health Sciences, Cumming School of Medicine, University of Calgary, Calgary, AB, Canada; 13 Division of Population Health and Applied Health Sciences, Faculty of Medicine, Memorial University, St. John's, NLFD, Canada; Mayo Clinic College of Medicine and Science, UNITED STATES OF AMERICA

## Abstract

**Background:**

The last 90 days of life are marked by high healthcare utilization in acute care settings, often conflicting with the preference to remain at home. The COVID-19 pandemic accelerated the adoption of virtual care, but its impact on healthcare utilization near the end-of-life remains unclear. This study assessed the association between physician-delivered virtual care use near the end-of-life and acute healthcare utilization, before and during the COVID-19 pandemic across four Canadian provinces.

**Methods:**

A retrospective population-based cohort study using linked health administrative data from January 1, 2018, to December 31, 2021, across British Columbia (BC), Alberta (AB), Ontario (ON), and Newfoundland & Labrador (NFLD). The study included 548,955 adult decedents who died within the study period. Virtual care use in the last 90 days of life, categorized by pre-pandemic and pandemic periods, was the primary exposure. Primary outcomes were rates of ED visits, hospitalizations, and in-hospital deaths during the last 90 days of life. Modified Poisson regression models were used to measure associations, adjusting for demographic and clinical characteristics.

**Results:**

Among the 548,955 decedents, virtual care utilization during the pandemic varied by province, ranging from 53% in NFLD to 78% in BC. During the pandemic, virtual care was associated with higher ED visits (adjusted rate ratios [aRateRs] ranging from 1.12 to 1.72) and hospitalizations (aRateRs: ranging from 1.01 to 1.59) in most provinces. Virtual care was linked to a higher risk of in-hospital death in AB (adjusted risk ratios [aRiskR]: 1.11; 95% CI: 1.08–1.14; P < 0.001) and ON (aRiskR: 1.04; 95% CI: 1.03–1.05; P < 0.001). Pre-pandemic, associations were weaker, with virtual care linked to lower in-hospital death rates in ON, AB and BC.

**Conclusion:**

Virtual care during the pandemic was linked to increased acute healthcare utilization, contrasting with pre-pandemic patterns when it appeared more selective and associated with fewer in-hospital deaths. Findings highlight the evolving role of virtual care and the need for region-specific policies to optimize end-of-life care delivery.

## Background

Healthcare use in the last 90 days of life rises substantially and is often delivered in acute care settings such as emergency departments and hospitals [1]. This may be inconsistent with patient wishes, since most people express a desire to remain at home with healthcare supports at the end of life [2–3].Community-based palliative care teams can help facilitate care at home, and they often rely on virtual care (e.g., telephone or video) to achieve this [4–7], or to identify people with unsustainable care needs and direct them to acute care.

In March 2020, Canadian jurisdictions introduced new physician fee codes to support virtual care delivery through telephone and video as restrictions on providing in-person healthcare became imperative to mitigate the risk of transmitting the SARS-CoV-2 virus [8]. Studies from Ontario have shown an association between virtual care and increased emergency department use [9,10], although this trend was not observed when virtual care was provided by family physicians [11]. However, these studies involved a single jurisdiction and may not reflect the diverse experiences of other jurisdictions, including other provinces and territories in Canada where universal healthcare exists, but policies and physician fee codes related to the use of virtual care near the end of life prior to the pandemic varied. Accordingly, we measured the association between the use of virtual care in the last 90 days of life and acute healthcare use in four Canadian provinces before and during the COVID-19 pandemic.

## Methods

This study is reported in accordance with guidelines for The Reporting of studies Conducted using Observational Routinely-collected health Data (RECORD) (S1 Appendix) [12]. Research ethics approval for this study was obtained in British Columbia (BC), Alberta (AB), and Newfoundland & Labrador (NFLD). In Ontario (ON), ICES (formerly the Institute for Clinical Evaluative Sciences) is a prescribed entity under Ontario’s Personal Health Information Protection Act (PHIPA). Projects that use data collected by ICES under section 45 of PHIPA are exempt from REB review. The use of the data in this project is authorized under section 45 and approved by ICES’ Privacy and Legal Office.

### Study design, setting and data sources

We conducted a retrospective population-based cohort study in four Canadian provinces, AB, BC, NFLD, and ON, using linked clinical and health administrative databases. These four provinces collectively constitute more than 66% of Canada’s diverse population of 41 million people, underscoring their significant impact on the nation’s demographic composition [13]. The datasets used in the study were linked using unique encoded identifiers and analyzed at the provincial level (S1 Table in S1 File).

### Study cohort

The study cohort included all adults (aged ≥18 years) in their last 90 days of life who died between January 1, 2018, and December 31, 2021, in the provinces of AB, BC, NFLD and ON. We identified all decedents during the study period and assigned their final 90 days of life as the exposure window, counting back from the date of death. People were excluded from the study if they were aged >105 years to minimize data errors or misclassification due to rare age-related outliers in administrative datasets. We also excluded patients missing age or sex data on the 90th day before their death (i.e., eligibility date), non-residents of AB, BC, NFLD or ON, ineligible for their provincial health insurance for ≥3 months in the previous year, resided in a nursing home, or were hospitalized throughout the entire 90 days before their death as they would not have had an opportunity to receive virtual care in the last 90 days of life.

### Use of virtual care near the EOL (Exposure)

The primary exposure was the first instance of the use of virtual care in the last 90 days of life, which served as the index date. The use of virtual care near the end of life was measured using a distinct set of specialized virtual care physician fee codes that were introduced in the four provinces in March 2020 (S2 Table in S1 File). Individuals who did not have any virtual care visits during the last 90 days of their life were categorized as the “unexposed” group and were randomly assigned an index date. We determined their index date (or pseudo-index date) and counted the ED and hospitalizations from that date to death. People were assigned to either the pre-pandemic or pandemic group based on their index date and date of death. Those with both an index and death date prior to March 14, 2020, were assigned to the pre-pandemic group. Conversely, a person whose index and death dates were on or after March 14, 2020 was assigned to the pandemic group. People who died <45 days before March 14, 2020 were assigned to the pre-pandemic group and people who died ≥45 days after were assigned to the pandemic group.

### Patient characteristics

We measured patients’ demographic and clinical variables, including age, sex, surname-based ethnicity, socioeconomic status, rural residence, and chronic health conditions, which were defined according to the Canadian Chronic Disease Surveillance System (CCDSS) criteria [14]. We also calculated the hospital frailty risk score, using a five-year look-back period from the index date. Additionally, we recorded each patient’s year and location of death, utilization of acute healthcare services (such as ED visits and hospitalizations), and use of virtual care in the year prior to the study index date. Ethnicity was classified using the SURNAMES algorithm, which applies validated Chinese and South Asian surname lists to the population under study [15]. According to this approach, individuals identified through these lists were categorized as Chinese or South Asian; all others were grouped under ‘general population’, which includes individuals of European descent and other groups not captured by the surname lists. This variable was used descriptively to assess potential differences in virtual care use. It was not included in analytic models due to its limited sensitivity and corresponding ICES policies restricting its use for such purposes.

### Outcomes

The primary outcomes were emergency department visits and hospitalization during the last 90 days of life, starting from the index date of the first virtual or in-person visit during this period. The secondary outcome was in-hospital death, measured as a binary outcome.

### Statistical analysis

We used different models to assess associations between virtual end-of-life care and primary outcomes during the last 90 days of life at the individual patient level within each province. For count-based outcomes, such as the number of ED visits and hospitalizations, we used a regular Poisson regression model, with an offset to account for different lengths of follow-up period, to estimate rate ratio (RateR). For in-hospital death, a binary outcome, we used a modified Poisson model to estimate risk ratio (RiskR).

All models were adjusted for potential confounding factors, including age, sex, rurality, neighbourhood income quintile, prevalent comorbidities, hospital frailty risk score, use of virtual care in the year prior to index date, ED visits and hospitalizations in the year prior to index date. The selection of these covariates was based on the clinical and research expertise of our team. All analyses were performed using SAS version 9.4 (SAS Institute, Cary, North Carolina). We did not measure the associated effects of virtual care in the last three months of life on other important end-of-life outcomes such as quality of life, symptoms, and healthcare use. We report balance diagnostics across baseline characteristics using standardized differences over statistical tests to assess balance between groups which are confounded with sample size. A standardized difference >0.10 was considered meaningful [16].

## Results

### Characteristics of the Study Cohort

**Fig 1:**
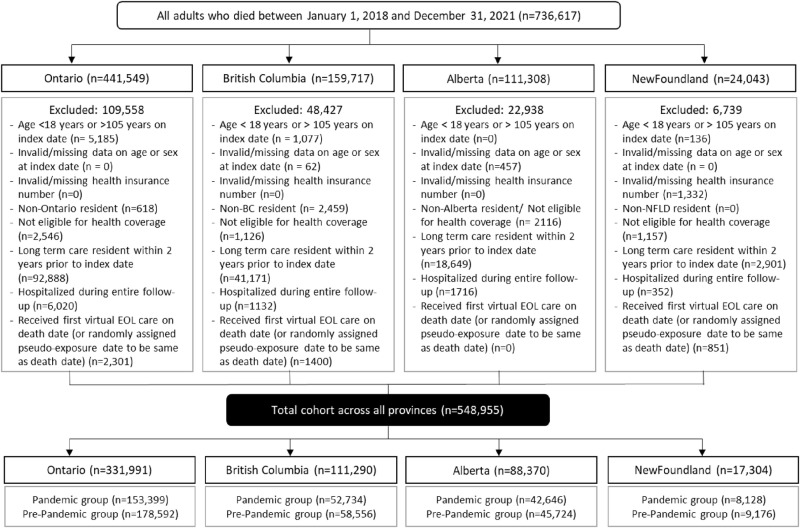
The figure illustrates how the study cohort was created and the distribution of patients across the provinces.

There were 548,955 adult decedents included in the study cohort (Fig 1). During the pandemic, the use of virtual care near the end of life varied across the four provinces: BC (78%, n = 41,043), AB (70%, n = 30,055), ON (69%, n = 106,631) and NFLD (53%, n = 4,296) (Table 1). Before the pandemic, BC and AB had higher rates of virtual EOL care (44% and 48%, respectively) compared to ON (8%) and NFLD (0.2%) (S3 Table in S1 File).

**Table 1:** Baseline characteristics of adults who died in Canada, in one of four provinces, during the COVID-19 pandemic period. Individuals were grouped by use of virtual care (exposed) compared to those who did not (unexposed) in the last 90 days of life.

	ONTARIO (n = 153,399)			NEW FOUNDLAND (n = 8,128)			BRITISH COLUMBIA (n = 52,734)			ALBERTA (n = 42,646)		
VARIABLE	Received virtual care (Exposed)	Did not received virtual care (Unexposed)	StdD	Received virtual care (Exposed)	Did not received virtual care (Unexposed)	StdD	Received virtual care (Exposed)	Did not received virtual care (Unexposed)	StdD	Received virtual care (Exposed)	Did not received virtual care (Unexposed)	StdD
N (%)	106,631 (69.5)	46,768 (30.5)		4,296 (52.9)	3,832 (47.1)		41,043 (77.8)	11,691 (22.2)		30,055 (70.5)	12,591 (29.5)	
Age, Mean ± SD	75.4 ± 14.4	72.3 ± 18.7	0.19	75.7 ± 12.8	75.7 ± 14.8	0	75.0 ± 14.7	65.4 ± 19.3	0.56	74.8 ± 15.5	63.2 ± 20.2	0.64
Female Sex, n (%)	48,952 (45.9)	19,343 (41.4)	0.09	2025 (47.1)	1722 (44.9)	0.04	18,029 (43.9)	3,777 (32.3)	0.24	13,998 (46.6)	4,226 (33.6)	0.27
Neighbourhood income quintile												
1 (Lowest)	25,263 (23.7)	14,155 (30.3)	0.15	1005 (23.4)	989 (25.8)	0.1	10,917 (26.6)	4,067 (34.8)	0.18	10,579 (35.2)	5,184 (41.2)	0.12
2	23,349 (21.9)	10,307 (22.0)	0	923 (21.5)	806 (21.0)	0.01	8,742 (21.3)	2,442 (20.9)	0.01	6,400 (21.3)	2,639 (21.0)	0.01
3	21,137 (19.8)	8,588 (18.4)	0.04	901 (21.0)	811 (21.2)	0	7,852 (19.1)	2,008 (17.2)	0.05	4,989 (16.6)	1,888 (15.0)	0.04
4	18,545 (17.4)	6,979 (14.9)	0.07	894 (20.8)	700 (18.3)	0.06	6,866 (16.7)	1,671 (14.3)	0.07	4,023 (13.4)	1,477 (11.7)	0.05
5 (Highest)	17,927 (16.8)	6,397 (13.7)	0.09	543 (12.6)	507 (13.2)	0	6,485 (15.8)	1,358 (11.6)	0.12	3,757 (12.5)	1,240 (9.9)	0.08
Missing	410 (0.4)	342 (0.7)	0.05	30 (0.7)	19 (0.5)	0.03	181 (0.4)	145 (1.2)	0.09	307 (1.0)	163 (1.3)	0.03
Surname based Ethnicity												
Chinese	3,199 (3.0)	1,025 (2.2)	0.05	4304^*^ (100)	3832^*^ (100)	0.01	2,245 (5.5)	645 (5.5)	0	676 (2.3)	283 (2.3)	0
General	100,639 (94.4)	44,995 (96.2)	0.09				37,438 (91.2)	10,739 (91.9)	0.02	28,909 (96.2)	12,134 (96.4)	0.01
South-Asian	2,767 (2.6)	735 (1.6)	0.07				1,360 (3.3)	307 (2.6)	0.04	470 (1.6)	173 (1.4)	0.02
Missing	26 (0.0)	13 (0.0)	0	--	--	--	--	--	--	0 (0.0%)	1 (0.01)	0.04
Rural residence, n (%)												
Yes	12,359 (11.6)	7,356 (15.7)	0.12	2394 (55.7)	2519 (65.7)	0	6,037 (14.7)	1,763 (15.1)	0.01	6,459 (21.5)	2,943 (23.4)	0.05
Missing	366 (0.3)	315 (0.7)	0.05	36 (0.8)	13 (0.3)	0.07	177 (0.4)	138 (1.2)	0.08	84 (0.3)	84 (0.7)	0.06
Alcohol and substance use disorder	3,923 (3.7)	2,846 (6.1)	0.11	97 (2.3)	101 (2.6)	0.03	974 (2.4)	768 (6.6)	0.20	1,885 (6.3)	1,571 (12.5)	0.21
Asthma	17,568 (16.5)	6,314 (13.5)	0.08	466 (10.9)	360 (9.4)	0.07	5,722 (13.9)	1,261 (10.8)	0.10	5,809 (19.3)	1,944 (15.4)	0.10
Cancer	27,868 (26.1)	4,429 (9.5)	0.45	1104 (25.7)	682 (17.8)	0.27	9,451 (23.0)	951 (8.1)	0.42	7,104 (23.6)	913 (7.3)	0.47
Cirrhosis	2,581 (2.4)	819 (1.8)	0.05	99 (2.3)	59 (1.5)	0.08	811 (2.0)	204 (1.7)	0.02	854 (2.8)	239 (1.9)	0.06
COPD	37,482 (35.2)	13,467 (28.8)	0.14	1526 (35.5)	1087 (28.4)	0.22	14,709 (35.8)	2,705 (23.1)	0.28	12,414 (41.3)	3,475 (27.6)	0.29
Dementia	14,337 (13.4)	7,368 (15.8)	0.07	530 (12.3)	791 (20.6)	0.32	3,946 (9.6)	759 (6.5)	0.11	5,119 (17.0)	947 (7.5)	0.29
Diabetes	41,817 (39.2)	14,640 (31.3)	0.17	1228 (28.6)	943 (24.6)	0.13	14,822 (36.1)	2,719 (23.3)	0.28	10,936 (36.4)	3,115 (24.7)	0.25
Heart failure	29,412 (27.6)	10,067 (21.5)	0.14	1250 (29.1)	912 (23.8)	0.17	12,185 (29.7)	1,755 (15.0)	0.36	9,627 (32.0)	2,019 (16.0)	0.38
Hypertension	79,345 (74.4)	29,862 (63.9)	0.23	3497 (81.4)	2708 (70.6)	0.36	29,156 (71.0)	5,514 (47.2)	0.50	22,799 (75.9)	6,778 (53.8)	0.47
Non-psychotic disorder	5,177 (4.9)	2,234 (4.8)	0	37^*^ (0.9)	34^*^ (0.89)	0.02	695 (1.7)	271 (2.3)	0.04	1,549 (5.2)	659 (5.2)	0.00
Psychotic disorder	648 (0.6)	495 (1.1)	0.05				342 (0.8)	285 (2.4)	0.13	331 (1.1)	302 (2.4)	0.10
Renal failure	22,410 (21.0)	7,284 (15.6)	0.14	773 (18.0)	529 (13.8)	0.16	8,663 (21.1)	1,548 (13.2)	0.21	6,329 (21.1)	1,327 (10.5)	0.29
Stroke	19,706 (18.5)	8,162 (17.5)	0.03	748 (17.4)	741 (19.3)	0.07	6,940 (16.9)	1,320 (11.3)	0.16	6,147 (20.5)	1,499 (11.9)	0.23
Hospital frailty risk score, n (%)												
0	12,425 (11.7)	3,381 (7.2)	0.15	1309 (30.5%)	1024 (26.7)	0.08	5,807 (14.1)	1,131 (9.7)	0.14	3,786 (12.6)	1,206 (9.6)	0.1
0.1 - 4.9	24,838 (23.3)	7,930 (17.0)	0.16	1159 (27.0%)	877 (22.9)	0.09	11,100 (27.0)	2,416 (20.7)	0.15	7,377 (24.6)	2,481 (19.7)	0.12
5.0 - 8.9	13,862 (13.0)	4,894 (10.5)	0.08	584 (13.6%)	520 (13.6)	0	5,362 (13.1)	1,092 (9.3)	0.12	3,997 (13.3)	1,011 (8.0)	0.17
9.0 +	23,596 (22.1)	9,590 (20.5)	0.04	816 (19.0%)	828 (21.6)	0.1	6,709 (16.3)	1,425 (12.2)	0.12	7,456 (24.8)	1,588 (12.6)	0.32
No hospitalizations	31,910 (29.9)	20,973 (44.8)	0.31	428 (10.0%)	583 (15.2)	0.2	12,065 (29.4)	5,627 (48.1)	0.39	7,439 (24.8)	6,305 (50.1)	0.54
Number of unique ED visits in past year^ⴕ^, Mean ± SD	1.4 ± 2.6	1.1 ± 3.1	0.09	2.5 ± 3.8	2.1 ± 3.5	0.11	1.4 ± 2.7	1.2 ± 3.2	0.08	1.4 ± 3.2	1.0 ± 3.0	0.12
Number of unique hospitalization episodes in past year, Mean ± SD	0.8 ± 1.3	0.5 ± 1.1	0.26	0.9 ± 1.4	0.6 ± 1.1	0.18	0.8 ± 1.3	0.5 ± 1.2	0.25	0.8 ± 1.3	0.4 ± 1.0	0.38

* Smaller categories have been aggregated/suppressed to protect participant confidentiality.

ⴕ Emergency Department visits that did not result in admission to hospital.

ED- Emergency Department; SD- Standard deviation; StdD- Standardized difference.

The baseline characteristics of patients who received virtual care near the end-of-life during the pandemic and pre-pandemic periods across provinces are reported in Table 1 and S3 Table in S1 File. During the pandemic period, patients who received virtual end-of-life care were generally older, more likely to be female, have higher rates of cancer and other chronic comorbid conditions like hypertension, heart failure, chronic obstructive pulmonary disease, and chronic kidney disease, and have a higher number of hospitalization episodes in the past year, compared to those who did not receive virtual care. A lower proportion of people who received virtual end-of-life care had alcohol and substance use disorders (Table 1). These observed trends were largely similar in the pre-pandemic period, but the magnitude of these differences was typically smaller (S3 Table, S1 Figure A and S1 Figure B in S1 File).

### Association of virtual near the EOL care and acute healthcare use

**Fig 2:**
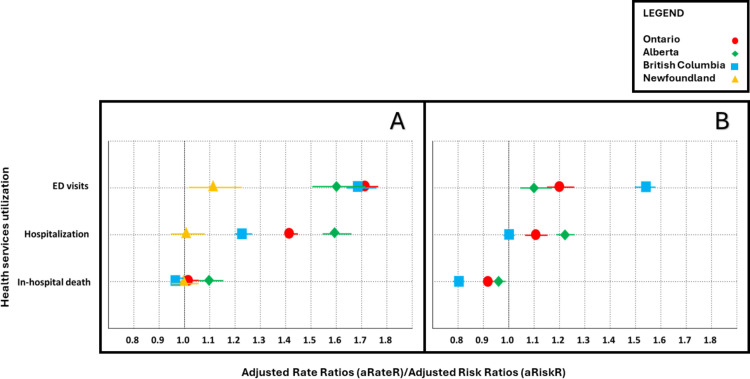
The Love plots illustrate the association between receipt of virtual care near EOL and acute health services utilization rates and location of death among adults, who either received (exposed) or did not receive (unexposed) virtual care. Panel A shows the association during the COVID-19 pandemic. Panel B displays the association in the pre-pandemic period. Red circles represent Ontario, Yellow triangles are for Newfoundland, Blue boxes for British Columbia, and Green diamonds for Alberta.

The use of virtual care near the EOL was associated with higher rates of acute healthcare use in AB, BC, and ON, but not in NFLD. The adjusted rate ratios and 95% CI for each outcome across all provinces are reported in Table 2. During the pandemic, virtual EOL care in AB was associated with a 60% higher rate of emergency department (ED) use and a 59% higher rate of hospitalizations (Table 2, Fig 2A). In BC, virtual EOL care was associated with a 69% higher rate of ED visits and 24% higher rate of hospitalizations. The magnitude of these associations was highest in ON with 72% higher rates of ED visits and 43% higher rates of hospitalization. In NFLD virtual EOL care was associated with a 12% higher rate of ED visits, but was not associated with hospitalization (Table 2, Fig 2A).

**Table 2:** Association between receipt of virtual care near end-of-life and acute health services utilization rates and location of death among adults, during the COVID-19 pandemic.

Outcome	ONTARIO		NEW FOUNDLAND		BRITISH COLUMBIA		ALBERTA	
	Crude rate	aRateR/ aRiskR^ⴕ^ (95% CI)	Crude rate	aRateR/ aRiskR (95% CI)	Crude rate	aRateR/ aRiskR (95% CI)	Crude rate	aRateR/ aRiskR (95% CI)
Emergency Department visits (mean ± SD)	Exposed: 0.5 ± 1.1 Unexposed: 0.3 ± 0.9	1.72^*^ (1.68, 1.76)	Exposed: 0.6 ± 1.0 Unexposed: 0.5 ± 1.1	1.12^*^ (1.03, 1.22)	Exposed: 0.7 ± 1.3 Unexposed: 0.4 ± 1.1	1.69^*^ (1.63, 1.75)	Exposed: 0.4 ± 1.1 Unexposed: 0.3 ± 0.8	1.60^*^ (1.50,1.71)
Hospitalization (mean ± SD)	Exposed: 0.8 ± 0.8 Unexposed: 0.5 ± 0.7	1.43^*^ (1.41, 1.45)	Exposed: 0.7 ± 0.7 Unexposed: 0.5 ± 0.6	1.01 (0.94, 1.09)	Exposed: 0.9 ± 0.8 Unexposed: 0.6 ± 0.7	1.24^*^ (1.20, 1.28)	Exposed: 0.8 ± 0.8 Unexposed: 0.4 ± 0.6	1.59^*^ (1.54,1.65)
In-hospital death N (%)	Exposed: 50,837 (47.7%) Unexposed: 20,347 (43.5%)	1.04^*^ (1.03, 1.05)	Exposed: 2347 (54.6%) Unexposed: 1854 (48.4%)	1.00 (0.94, 1.05)	Exposed: 17,963 (43.8) Unexposed: 4,425 (37.8)	0.97 (0.94, 1.00)	Exposed: 14,643 (48.7%) Unexposed: 4,717 (37.5%)	1.11^*^ (1.08,1.14)

Comparison between exposed and unexposed group.

* p < 0.01.

ⴕ Adjusted Rate Ratios (aRateRs) are reported for count outcomes (ED visits and hospitalizations), estimated using Poisson regression. Adjusted Risk Ratio (aRiskR) is reported for the binary outcome (in-hospital death), estimated using modified Poisson regression. Models were adjusted for age, sex, rurality, neighbourhood income quintile, prevalent comorbidities, hospital frailty risk score, any use of virtual care in the year prior to index date and acute healthcare system use (such as ED days and hospitalization) in the year prior to index date.

Before the pandemic, the associations were similar but less pronounced. Virtual EOL care in AB was associated with a 10% higher rate of ED visits and a 22% higher rate of hospitalization. In BC, virtual EOL care was associated with a 54% higher rate of ED use but no significant association with hospitalizations. In ON, these rates were 20% and 11% higher, respectively. We were unable to model associations in NFLD due to small sample size (Table 3, Fig 2B).

**Table 3:** Association between receipt of virtual care near end-of-life and acute health services utilization rates and location of death among adults, prior to COVID-19 pandemic.

Outcome	ONTARIO		NEW FOUNDLAND		BRITISH COLUMBIA		ALBERTA	
	Crude rate	aRateR/ aRiskR (95% CI)	Crude rate	aRateR/ aRiskR (95% CI)	Crude rate	aRateR/ aRiskR (95% CI)	Crude rate	aRateR/ aRiskR (95% CI)
Emergency Department visits (mean ± SD)	Exposed: 0.4 ± 1.2 Unexposed: 0.3 ± 0.8	1.20^*^ (1.16, 1.24)	Exposed: 0.9 + 1.8 Unexposed: 0.4 ± 0.9	–	Exposed: 0.7 ± 1.3 Unexposed: 0.5 ± 1.0	1.54* (1.50, 1.58)	Exposed: 0.4 ± 1.0 Unexposed: 0.3 ± 0.8	1.10^*^ (1.04,1.17)
Hospitalization (mean ± SD)	Exposed: 0.6 ± 0.7 Unexposed: 0.5 ± 0.7	1.11^*^ (1.08, 1.14)	Exposed: 0.7 ± 0.7 Unexposed: 0.6 ± 0.6	–	Exposed: 0.9 ± 0.8 Unexposed: 0.8 ± 0.8	1.00 (0.98, 1.02)	Exposed: 0.7 ± 0.8 Unexposed: 0.5 ± 0.6	1.22^*^ (1.19,1.25)
In-hospital death N (%)	Exposed: 6,246 (44.8%) Unexposed: 82,260 (50.0%)	0.92^*^ (0.90, 0.94)	Exposed: 8 (47.1%) Unexposed: 5258 (57.4%)	–	Exposed: 11,211 (43.7%) Unexposed: 16,556 (50.4%)	0.81^*^ (0.79, 0.82)	Exposed: 11,519 (51.8%) Unexposed: 11,089 (47.2%)	0.97^*^ (0.95,0.99)

Comparison between exposed and unexposed group.

* p < 0.01.

ⴕ Adjusted Rate Ratios (aRateRs) are reported for count outcomes (ED visits and hospitalizations), estimated using Poisson regression. Adjusted Risk Ratio (aRiskR) is reported for the binary outcome (in-hospital death), estimated using modified Poisson regression. Models were adjusted for age, sex, rurality, neighbourhood income quintile, prevalent comorbidities, hospital frailty risk score, any use of virtual care in the year prior to index date and acute healthcare system use (such as ED days and hospitalization) in the year prior to index date.

### Association of virtual care near the EOL and in-hospital death

The use of virtual care near the EOL during the pandemic was associated with a higher risk of in-hospital death in AB (aRiskR 1.11, 95% CI 1.08 to 1.14) and ON (aRiskR 1.04, 95% CI 1.03 to 1.05), but not in BC or NFLD (Table 2, Fig 2A). In contrast, before the pandemic, receiving virtual care near the EOL was associated with a lower risk of in-hospital death across all the provinces: 8% in ON (aRiskR 0.92, 95% CI 0.90 to 0.94), 19% in BC (aRiskR 0.81, 95% CI 0.79 to 0.82) and 3% (aRiskR 0.97, 95% CI 0.95 to 0.99) in AB (Table 3, Fig 2B).

## Discussion

This population-based cohort study of 548,955 Canadians found that use of virtual care near the end of life during the pandemic and pre-pandemic periods was associated with increased rates of acute healthcare use across four geographically diverse provinces that comprise 66% of the total population of Canada. Virtual care was also associated with an increased risk of dying in hospital in two out of four provinces during the pandemic (ON, AB), but was associated with a reduced risk of death in the pre-pandemic period in all provinces. The magnitudes of these associations varied by province and pandemic period. However, they did not appear to vary according to a province’s pre-pandemic levels of virtual care use near the end-of-life.

Health systems are increasingly focused on delivering high-value care that achieves desired health outcomes at the lowest possible cost. End-of-life care often involves high healthcare costs due to frequent emergency department visits and hospitalizations. However, most people prefer to receive end-of-life care at home and also to die there. As health systems transition out of the pandemic and virtual end-of-life care becomes more accessible and commonly utilized, it is crucial to assess its value for both the users and the payers who reimburse it. This may be especially important as some jurisdictions have begun to limit access to certain types of virtual care. This assessment will inform ongoing coverage policies at a system level. Our research sheds light on the patterns of acute healthcare usage and the locations of death for individuals who did and did not receive virtual end-of-life care across Canada, highlighting regional variations among provinces with distinct health systems, organization of healthcare delivery, and resources. Some of this variation may be related to differences in regional policies that direct when and for whom to use virtual care. These findings can inform decision-makers on how best to utilize virtual care at the end of life. For example, in regions where virtual care is associated with lower rates of acute healthcare usage and where patients are more likely to die at home, policies could be implemented to encourage the use of virtual care, perhaps through enhanced reimbursement models or targeted funding to support infrastructure. Conversely, in areas where virtual care does not seem to reduce acute care usage or improve patient outcomes, policy levers such as reimbursement adjustments or the introduction of stricter eligibility criteria might be used to ensure that virtual care is applied more selectively.

The higher use of virtual end-of-life care during the pandemic in BC (78%) and AB (70%), compared to ON (69%) and NFLD (53%), may reflect regional differences in healthcare infrastructure and pre-pandemic care delivery. BC and AB had already integrated virtual end-of-life care into their healthcare systems more extensively before the pandemic (44% and 48%, respectively), which likely facilitated care delivery and outcomes during rapid escalation of virtual care use during the pandemic. In contrast, the lower pre-pandemic use of virtual care near the end-of-life in ON and NFLD (8% and 0.2%, respectively), meant that these regions had to rapidly adapt to a new form of care delivery, potentially influencing the observed associations with acute healthcare use.

The rapid increase in virtual care use in regions like ON and NFLD, where it was previously underutilized, may have led to higher rates of ED visits and hospitalizations, as providers and patients adjusted to this new modality of care. Additionally, the differences in sociodemographic and clinical characteristics among virtual end-of-life care users versus non-users may indicate that those who adopted virtual care may have had different healthcare needs which could partially explain the varied associations with acute healthcare usage and location of death. For instance, in regions where virtual care was already established, the lower observed hospitalization rates may reflect more effective management of end-of-life care needs virtually, reducing the need for acute care interventions. Conversely, in areas with less pre-pandemic virtual care experience, the transition may have been associated with higher acute care use as patients and providers navigated the new system. It may also be related to differences in how and for whom virtual care was used. These regional differences in virtual care adoption and the underlying healthcare needs of patients highlight the importance of tailoring virtual care policies to ensure they meet the specific needs of each population.

The higher associated risk of in-hospital death in AB and ON contrasts with the pre-pandemic period, where virtual EOL care was associated with a reduced risk of in-hospital death across all provinces. The pandemic likely disrupted usual care practices, increased patient acuity, and strained healthcare resources, potentially leading to higher in-hospital death rates despite the provision of virtual care outside hospital. The lack of increased risk observed in BC during the pandemic bears further study, which may indicate a more robust adaptation to virtual EOL care than other provinces, perhaps due to their pre-existing higher rates of usage and other contextual factors. These variations underscore the importance of understanding regional disparities in the adoption and integration of virtual care services across a large, geographically and socioeconomically diverse country like Canada.

Importantly, we could not distinguish between deaths due to expected chronic decline versus sudden or acute events. This may affect interpretation, as the need for hospital-based care may differ substantially between these trajectories. The observed association between virtual care and acute care use may reflect either appropriate escalation due to unmet home-based care needs or limitations in virtual care assessments resulting in precautionary hospital transfers. Without detailed clinical context or patient-reported data, we could not determine the appropriateness or patient preferences for these transitions.

Further, we did not have access to outcomes such as patient satisfaction or markers of potentially inappropriate care (e.g., ICU admissions, mechanical ventilation). These measures would be valuable for assessing the quality of care and goal-concordance and should be prioritized in future research.

Future studies should also consider stratifying outcomes by patient frailty, presence of dementia, advanced illness stage (e.g., metastatic cancer or end-stage organ failure), and prior palliative care involvement. These subgroups may have differing trajectories and care needs and are less likely to benefit from acute hospital interventions. Such stratification may clarify which patients are most appropriately supported through virtual care at the end of life.

### Strengths and limitations

This study’s strengths include the use of a large, diverse cohort from four Canadian provinces, which represent over 66% of the national population, enhancing the generalizability of the findings. The multi-provincial approach allows for a comparative analysis across regions with varying healthcare infrastructures, providing a more comprehensive understanding of virtual care near the end-of-life and its association with healthcare utilization. The study’s robust statistical analysis, with adjustments for key confounders, further strengthens the reliability of the results.

Our study is limited by the use of administrative data that do not measure patient preferences for care, specific care needs, and care quality. For example, while most individuals express a preference to die at home [2,17], this preference is not measured in administrative data to ascertain concordance with these goals. Second, we measured use of virtual care in the last 90 days of life, and not specifically virtual palliative care. We were not able to measure cause of death or how they died so we cannot be sure why people received virtual end-of-life care visits. Nonetheless, our findings are broadly representative across various disease categories with different healthcare needs in the last 90 days of life, and is commonly used in end-of-life research to avoid reliance on physician prognostication and standardizes the lookback period relative to death [18,19]. Third, we did not measure the associated effects of virtual care in the last three months of life on other important end-of-life outcomes such as quality of life, symptoms, and healthcare use. The significant increase in use of virtual care does not fully account for the quality of virtual care provided or associated patient outcomes. The use of acute healthcare services in our study may reflect both an underlying need for these settings and potential gaps in access to home-based care or individual preferences for care settings. Fourth, the broad definition of virtual care covered various delivery modalities [20] (e.g., telephone, videoconference) (S2 Table in S1 File) and settings (e.g., family physicians, specialists), this may obscure the specific effects of different types of virtual care on various outcomes. Previous studies suggest that patients whose family physicians provided a high proportion of virtual care during the COVID-19 pandemic did not experience higher emergency department visit rates, whereas those attending virtual walk-in clinics were more likely to visit the emergency department [9,11]. Fifth, our study focused specifically on physician-delivered virtual care. However, it is important to recognize that virtual end-of-life care can be delivered by a range of clinicians, including nurses, social workers, physiotherapists, occupational therapists, and spiritual care providers, whose services were not captured in our analysis. Furthermore, the administrative data did not allow us to distinguish the clinical specialty of the provider (e.g., generalist vs. palliative care specialist) or the intent behind the virtual visit. As a result, we could not assess whether the associations observed were influenced by the nature or intent of the virtual care received, which limits the interpretability of our findings.

In addition, we lacked data on enrollment in home hospice or community palliative care programs, which may independently influence healthcare utilization and outcomes. Patient preferences for cardiopulmonary resuscitation (e.g., Do Not Resuscitate or Do Not Intubate) were also unavailable, limiting our ability to assess how these preferences may have influenced the likelihood of an in-hospital death. We also could not distinguish between deaths due to expected chronic decline versus sudden or acute events. This may affect interpretation, as the need for hospital-based care may differ substantially between these trajectories.

We categorized virtual care exposure based on receipt of at least one visit during the last 90 days of life. The frequency of visits and its association with the outcomes was not measured, but remains an important area for future research, as it may shed light on illness trajectories, care needs, or care quality.

Finally, our study was limited to four provinces with comprehensive, linkable health administrative data and timely access to virtual care billing codes. While these provinces represent the largest, and most ethnically diverse majority of the Canadian population, our findings may not generalize to other provinces or territories.

The COVID-19 pandemic significantly altered healthcare delivery, raising questions about the generalizability of our findings to future contexts [21–23]. However, the consistency of our findings across pre-pandemic and pandemic periods enhances our confidence in these associations. The association of virtual care near the end-of-life with acute healthcare use in the post-pandemic era bears further study.

## Conclusion

Use of virtual care near the end of life during the COVID-19 pandemic was associated with increased rates of acute healthcare use across four Canadian provinces, with notable regional variation in the magnitude of these associations. These findings suggest the need for region-specific health policies to optimize the use of different modalities of end-of-life care for the right patient, at the right time and that are contextually relevant to the health services available in each region.

### Site-specific statements for data sharing

ON data: The dataset from this study is held securely in coded form at ICES. While data sharing agreements prohibit ICES from making the dataset publicly available, access may be granted to those who meet pre-specified criteria for confidential access, available at www.ices.on.ca/DAS. The full dataset creation plan and underlying analytic code are available from the authors upon request, understanding that the computer programs may rely upon coding templates or macros that are unique to ICES and are therefore either inaccessible or may require modification. Additional requests may be sent to the Data Analytic Services at ICES using the following email address: das@ices.on.ca

BC data: Access to data provided by the Data Stewards is subject to approval but can be requested for research projects through the Data Stewards or their designated service providers. The following data sets were used in this study: Vital Events Deaths, Home and Community Care, PharmaNet, National Ambulatory Care Reporting System, Medical Services Plan (MSP) Payment Information File, Discharge Abstract Database (Hospital Separations), and Consolidation File (MSP Registration & Premium Billing). You can find further information regarding these data sets by visiting the PopData project webpage at: https://my.popdata.bc.ca/project_listings/22-050/collection_approval_dates. All inferences, opinions, and conclusions drawn in this publication are those of the author(s), and do not reflect the opinions or policies of the Data Steward(s).

AB data: Data was extracted from the Alberta Health Services Enterprise Data Warehouse with support provided by the Alberta Strategy for Patient Oriented Research Support Unit (AbSPORU) which is funded by CIHR, Alberta Innovates, University Hospital Foundation, University of Alberta, University of Calgary and Alberta Health Services. This study is based in part on data provided by Alberta Health and Alberta Health Services. The interpretation and conclusions contained herein are those of the researchers and neither the Government of Alberta nor Alberta Health Services expressed any opinion in relation to this study.

NFLD data: Data for this project was extracted and analyzed by Data and Information Requests, Newfoundland and Labrador Health Services (NLHS) – Digital Health. This study is partially supported by data and analytical services provided by NLHS Digital Health. All inferences and conclusions in this publication are those of the author(s) and do not necessarily reflect the opinions/policies of NLHS.

## Supporting information

S1 File **S1 Table.** Description of patient characteristics. **S2 Table.** Provincial Virtual Care Fee Codes. **S3 Table:** Baseline characteristics of adults who died in Canada. **S1 Figure A:** The Love plot illustrates the standardized differences in baseline characteristics across the four Canadian provinces among adults who died and either received (exposed) or did not receive (unexposed) virtual end-of-life care. Figure A shows the standardized differences during the COVID-19 pandemic. Red circles represent Ontario, Yellow triangles are for Newfoundland, Blue boxes for British Columbia, and Green diamonds for Alberta. **S1 Figure B:** The Love plot illustrates the standardized differences in baseline characteristics across the four Canadian provinces among adults who died and either received (exposed) or did not receive (unexposed) virtual end-of-life care. Figure B displays the differences in the pre-pandemic period. Red circles represent Ontario, Yellow triangles are for Newfoundland, Blue boxes for British Columbia, and Green diamonds for Alberta.
(DOCX)

S1 Appendix RECORD checklist. (DOCX)
