# Supplementary material for: Association of physician-delivered virtual care near the end of life with healthcare use outcomes: A national population-based study of Canadians
Source: PLoS One. 2025 Jun 3;20(6):e0324898. doi: 10.1371/journal.pone.0324898 (PMC12133171; doi:10.1371/journal.pone.0324898)
Supplement: S1 File — S1 Table. Description of patient characteristics. S2 Table. Provincial Virtual Care Fee Codes. S3 Table: Baseline characteristics of adults who died in Canada. S1 Figure A: The Love plot illustrates the standardized differences in baseline characteristics across the four Canadian provinces among adults who died and either received (exposed) or did not receive (unexposed) virtual end-of-life care. Figure A shows the standardized differences during the COVID-19 pandemic. Red circles represent Ontario, Yellow triangles are for Newfoundland, Blue boxes for British Columbia, and Green diamonds for Alberta. S1 Figure B: The Love plot illustrates the standardized differences in baseline characteristics across the four Canadian provinces among adults who died and either received (exposed) or did not receive (unexposed) virtual end-of-life care. Figure B displays the differences in the pre-pandemic period. Red circles represent Ontario, Yellow triangles are for Newfoundland, Blue boxes for British Columbia, and Green diamonds for Alberta. (DOCX) [file pone.0324898.s001.docx]

**S1 Table**: Description of patient characteristics, variables and databases used to capture them. Database access and linkage were facilitated by Health Data Research Network of Canada (<https://www.hdrn.ca/en/dash/>).

| **Characteristics** | **Variables** | **Databases** |
| --- | --- | --- |
| **Demographics** | - Age, sex, neighbourhood income quintile, immigration status, ethnicity (using postal code and last name), rurality, education level (using postal code) | - Discharge Abstract Database (DAD) - National Ambulatory Care Reporting System (NACRS) - Immigration, Refugees and Citizenship Canada (IRCC) + Longitudinal Immigration Database (IMDB) - Postal code Conversion File (PCCF) - British Columbia Central Demographics File/Consolidation file - Alberta Population Registry - Registered Persons Database (RPDB) - Newfoundland Client Registry |
| **Comorbidities** | - Heart failure, chronic obstructive pulmonary disease, diabetes, cancer, dementia, end-stage renal disease, cirrhosis, stroke, hypertension | - Discharge Abstract Database (DAD) |
| **Comorbidity Indices** | - Charlson index, Hospital frailty risk score | - Discharge Abstract Database (DAD) |
| **Death Data** | - Year of death; location of death | - Vital Statistics - Death Database (VSDD) - Discharge Abstract Database (DAD) |
| **Provincial Physician Billing** | - Virtual care | - Alberta Practitioner Claims - British Columbia Medical Services Plan Payment Information (MSP) & Premium Billing (Consolidation file) - Manitoba Medical Claims / Medical Services - New Brunswick Physician Billing - Newfoundland & Labrador Medical Care Plan (MCP) Fee-for-Service Physician Claims Database - Ontario Health Insurance Plan Claims Database (OHIP) - Saskatchewan Physician Services Claims File |
| **Healthcare Use (Last 90 days of life)** | - Emergency Department visits - Hospital days - LTC use (for exclusion criteria) | - Discharge Abstract Database (DAD) - National Ambulatory Care Reporting System (NACRS) - Continuing Care Reporting System (CCRS) |

**S2 Table: Provincial Virtual Care Fee Codes.** Codes presented in **red font** are “direct patient” care codes and are included in the primary analysis. Codes that are presented in **black font** are “provider to provider” codes.

| **Billing codes** | **Description** | **Notes** |
| --- | --- | --- |
| **ALBERTA** | **Alberta Practitioner Claims** | |
| **Pre-Pandemic** | | |
| **03.05JR** | Physician Telephone Call Directly to Patient to Discuss Test Results. | |
| **03.01S** | Physician to Patient Secure Electronic Communication. | |
| **03.01T** | Physician to Patient Secure Videoconference. | |
| **03.01NG / 03.01NH /**  **03.01 NI** | Patient care advice to paramedic - prehospital patch, Mobile Integrated Healthcare Unit paramedic, assisted living/designated assisted living and lodge staff, active treatment facility worker for hospital in-patient, long term care worker for patients in a long-term care facility, nurse practitioner, hospice worker, home care worker, midwife or public health nurse, provided via telephone or  other telecommunication methods, in relation to the care and treatment of a patient | |
| **TELE / TELES** | TELEHEALTH. This modifier is used to indicate telehealth services. The TELES modifier may only be claimed when providing services at a regional telehealth site or registered Health Canada Telehealth location. The use of SKYPE or any other secure video transfer system may not be claimed using the TELES modifier. Only services provided using regional or Health Canada telehealth equipment may be claimed using the TELES modifier. The TELES modifier is NOT to be claimed for services provided via telephone. | |
| **03.01C** | Telehealth assistance service. May only be claimed if the physician is required to be  present at the referring site to assist with essential physical assessment without which the consultant service would be ineffective. | |
| **03.05JH** | Family conference via telephone, in regard to a community patient. | |
| **Pandemic** | | |
| **03.01AD** | Advice to Patient or their Agent via Telephone, Email and Videoconference including Virtual Care during a Viral Epidemic. | Mar 12, 2020 |
| **03.03CV** | Assessment provided by General Practitioners (GP) and Specialists via telephone or secure videoconference; 10+ minutes physician: patient direct time. | Mar 17, 2020;  Made permanent Jun 9, 2020 |
| **03.03FV** | Follow up assessments (visits), for referred patients only, provided by specialists via telephone or secure videoconference; 10+ minutes physician: patient direct time. |  |
| **03.08CV** | Comprehensive consultations provided via telephone or secure videoconference; consultation requirements apply. |  |
| **08.19CX** | Comprehensive psychiatric consultation provided via telephone or secure videoconference; consultation requirements apply. |  |
| **08.19CV** | Psychotherapy and other psychiatric services (such as group therapy) provided via telephone or secure videoconference by a Psychiatrist or a Generalist in Mental Health. |  |
| **08.19CW** | Psychotherapy and other psychiatric services, chronic pain management or palliative care (such as group therapy) provided via telephone or secure videoconference by a GP or Pediatrician, per full 15 minutes. |  |
| **BRITISH**  **COLUMBIA** | **Medical Services Plan (MSP) & Premium Billing (Consolidation**  **file), Medical Services Plan Payment Information (MSP)** | |
| **Pre-Pandemic** | | |
| **13017, 13018, 13037,**  **13038** | Telehealth visit | |
| **P13036, P13037, P13038** | Telehealth GP in-office consultation, visit or counselling | |
| **P13016, P13017, P13018** | Telehealth GP out-of-office consultation, visit or counselling | |
| **13020** | Telehealth FP assistant - physical assessment requested by receiving specialist | |
| **PG14076** | FP patient telephone management fee (directly with patient or patient's medical representative) | |
| **PG14078** | FP email / text / telephone medical advice relay fee (directly with patient or patient's medical representative) | |
| **PG14077** | FP conference with allied care provider and/or physician (telephone, videoconferencing or in-person) | |
| **PG14276** | Telephone management encounter code for physicians on alternate payment/funding models | |
| **PG33250** | Virtual communication with patient, or representative/family, for medically pertinent matters | |
| **P32370, P32372, P32376,** | Telehealth consultation or repeat/limited consultation, directive care, subsequent | |
| **P32377, PG32367,** | office visit or follow-up visit | |
| **P32378** |  | |
| **33270, 33271, 33272,** | Telehealth consultation, complex consultation, repeat or limited consultation, | |
| **33276, 33277, 33278** | directive care, subsequent office visit or subsequent hospital visit (General Internal | |
|  | Medicine specialists only) | |
| **33110, 33112, 33114,** | Telehealth consultation, repeat or limited consultation, prolonged visit for | |
| **33106, 33107, 33108** | counselling, directive care, subsequent office visit, subsequent hospital visit | |
|  | (Cardiology specialist) | |
| **33360, 33362, 33366,** | Telehealth consultation, repeat or limited consultation, prolonged visit for | |
| **33367, 33368** | counselling, directive care, subsequent office visit, subsequent hospital visit | |
|  | (Gastroenterology specialist) | |
| **33470, 33472, 33421,** | Telehealth consultation, repeat or limited consultation, comprehensive geriatric | |
| **33422, P33473, P33474,** | consultation, geriatric reassessment, comprehensive cognitive consultation, geriatric | |
| **P33423, P33424, 33476,** | reassessment to comprehensive consultation, complex consultation, repeat or | |
| **P33426, 33477, P33427,** | limited complex consultation, directive care, comprehensive or complex directive | |
| **33478, P33428** | care, subsequent office visit, comprehensive or complex subsequent office visit, | |
|  | subsequent hospital visit, comprehensive or complex subsequent hospital visit | |
|  | (Geriatrics specialist) | |
| **33570, 33572, 33577** | Telehealth consultation, repeat or limited consultation, subsequent office visit (Hematology and Oncology) | |
| **33630, 33632, 33636,** | Telehealth consultation, repeat or limited consultation, directive care, subsequent | |
| **33637, 33638** | office visit, subsequent hospital visit (Infectious diseases) | |
| **33730, 33732, 33736,** | Telehealth consultation, repeat or limited consultation, directive care, subsequent | |
| **33737, 33738** | office visit, subsequent hospital visit (Nephrology) | |
| **32110, 32112, 32114,** | Telehealth consultation, repeat or limited consultation, counseling, directive care, | |
| **32106, 32107, 32108** | subsequent office visit, subsequent hospital visit (Respirology) | |
| **31110, 31112, 31106,** | Telehealth consultation, repeat or limited consultation, directive care, subsequent | |
| **31107, 31108** | office visit, subsequent hospital visit (Rheumatology) | |
| **00470, 00471, 00476,** | Telehealth consultation, repeat or limited consultation, directive care, subsequent | |
| **00477, 00478** | office visit, subsequent hospital visit (Neurology) | |
| **60610, 60613, 60625,** | Telehealth consultation, repeat or limited consultation, directive care, subsequent | |
| **60614, 60608, 60624,** | office visit, subsequent hospital visit, patient management conference (Psychiatry) | |
| **00624, 60645** |  | |
| **70070, 70072, 70077,** | Telehealth consultation, repeat or limited consultation, directive care, subsequent | |
| **70078, 70076, 70080,** | office visit, subsequent hospital visit (General surgery) | |
| **70087** |  | |
| **08070, 08072, 08077,** | Telehealth consultation, repeat or limited consultation, subsequent office visit, | |
| **08078** | subsequent hospital visit (Urology) | |
| **83070** | Telehealth interventional radiology consultation | |
| **P13012** | Hospital at home family physician conference with allied care provider and/or physician (telephone, videoconferencing or in-person) | |
| **13000** | Telephone advice to a community health representative in First Nation's communities | |
| **13005** | Advice about a patient in community care (telephone, fax, or written form) | |
| **PG14018, PG14019** | FP telephone advice from a specialist or family physician with consultative expertise (urgent, within 2 hours) | |
| **PG14021, PG14022, PG14023** | FPs with consultative expertise telephone or video advice (within 2 hours, within 1 week, management / follow-up) | |
|  |  | |
| **PG10001, PG10002, PG10003** | Specialist advice for patient management (phone, video technology or face-to-face)  - Urgent, Within 7 days, Follow-up | |
| **PG10005, PG10006** | Specialist email advice for patient management (7 days, follow-up) | |
| **PG10004** | Multidisciplinary conferencing for complex patients (face-to-face, telephone or video technology communication) | |
| **Pandemic** | | |
| **T13706** | FP Delegated Patient Telehealth Management Fee (similar to G14076) | Mar 27, 2020 |
| **T13707** | FP Email/Text/Telephone Medical Advice Relay or ReRX Fee (similar to G14078) |  |
| **T13708** | FP COVID-19 Communication With Specialist and/or Allied Care Provider (a new fee code) |  |
| **T10008** | Urgent Specialist COVID-19 Advice Fee (similar to G10001) |  |
| **T10007** | Specialist Email/Text/Telephone Medical Advice Relay or ReRX Fee (a new fee code) |  |
| **T10000** | Urgent Specialist Advice on patient with previous visit/service fee (similar to G10001) | Apr 15, 2020 |
| **T10009** | Non-Urgent Specialist Advice on patient with previous visit/service fee (similar to G10002) |  |
| **T13236, T13436, T13536, T13636, T13736, T13836** | Telehealth GP Consultation (Age 0-1, 2-49, 50-59, 60-69, 70-79,  80+ respectively) | Jun 1, 2020 |
| **T13237, T13437, T13537, T13637, T13737, T13837** | Telehealth GP Visit (Age 0-1, 2-49, 50-59, 60-69, 70-79, 80+  respectively) |  |
| **T13238, T13438, T13538, T13638, T13738, T13838** | Telehealth GP Individual counselling for a prolonged visit for counselling (Age 0-1, 2-49, 50-59, 60-69, 70-79, 80+ respectively) |  |
| **NEWFOUNDLAND AND LABRADOR** | **Medical Care Plan (MCP) Fee-for-Service Physician Claims Database** | |
| **Pre-Pandemic** | | |
| **520** | 2-way collaborative conferencing (telephone or in person) between FP and at least one primary health care provider (with or without patient) | |
| **521** | Patient care telephone for communication between FP and the patient | |
| **54166, 54167** | Anaesthesiologist initiation or maintenance of patient controlled analgesia (PCA) by visits or telephone consultation. | |
| **Pandemic** | | |
| **50000** | Pandemic Virtual Care Assessment (telephone or patient videoconferencing); effective August 17, 2020, this fee code is restricted to use by family physicians | Mar 13, 2020 |
| **253** | Consultation | Aug 12, 2020  (retroactive to July 17, 2020) |
| **254** | Reassessment |  |
| **255** | Anesthesiology, pre-anesthetic clinic assessment |  |
| **256** | Psychiatric care, per half-hour or major part thereof |  |
| **257-264** | Psychotherapy codes (Individual, Group, Case consultation Diagnostic interview with parent/guardian) |  |
| **ONTARIO** | **Ontario Health Insurance Plan Claims Database (OHIP)** | |
| **Pre-Pandemic** | | |
| **B099A** | Tracking Code | |
| **B100A** | First Telemedicine Patient Encounter premium | |
| **B101A** | First Cancelled/Missed Telemedicine Patient Encounter premium | |
| **B102A** | First Technical Difficulties Abandoned Patient Encounter premium | |
| **B200A** | Subsequent Telemedicine Patient Encounter premium | |
| **B201A** | Subsequent Missed/Cancelled Telemedicine Patient Encounter premium | |
| **B202A** | Subsequent Technical Difficulties Abandoned Patient Encounter premium | |
| **K708, K709, K710** | Multidisciplinary cancer conference | |
| **K730, K731, K734, K735,** | Physician to physician telephone consultation | |
| **G077** | Geriatric telephone support (for a caregiver of a patient with dementia) | |
| **K480** | Physician to allied professional telephone consultation | |
| **G382, G388** | Monthly telephone supervision of chemotherapy or special oral chemotherapy | |
| **G064** | Management and supervision of outpatient continuous nerve block infusion | |
| **G511** | Telephone management regarding a patient receiving palliative care at home | |
| **G512** | Palliative care case management fee | |
| **Pandemic** | | |
| **K080** | Minor assessment of a patient by telephone or video or advice or information by telephone or video to a patient’s representative regarding health maintenance, diagnosis, treatment and/or prognosis. | Mar 14, 2020  - Sep 30,  2021  (*Palliative care coverage from Mar 14,  2021 - Sep  30, 2021) |
| **K081** | 1. Intermediate assessment of a patient by telephone or video, or advice or information by telephone or video to a patient’s representative regarding health maintenance, diagnosis, treatment and/or prognosis, if the service lasts a minimum of 10 minutes; or 2. Psychotherapy, psychiatric or primary mental health care, counselling or interview conducted by telephone or video, if the service lasts a minimum of 10 minutes. |  |
| **K082** | Psychotherapy, psychiatric or primary mental health care, counselling or interview conducted by telephone or video per unit (unit means half hour or major part thereof). | Mar 14, 2020 |
| **K083** | Specialist Consultations and Visits by telephone or video. |  |
| **K084** | Premium equivalent for virtual care. | Oct 1, 2020 |
| **K087** | Minor assessment of an uninsured by telephone or video. | Mar 14, 2020 |
| **K088** | Intermediate assessment of an uninsured patient including psychotherapy by telephone or video. |  |
| **K089** | Psychotherapy, psychiatric or mental health counselling by telephone or video for an uninsured patient |  |
| **B203A** | Synchronous video visits with a patient in the home or another location of their choice (i.e. the patient is not at a patient host site). |  |
| **KXXX** | Virtual Palliative Care Codes (to be introduced May 1, 2021) | May 1, 2021 |

**S3 Table: Baseline characteristics of adults who died in Canada, in one of four provinces, during the pre- COVID-19 pandemic period. Individuals were grouped by use of virtual care (exposed) compared to those who did not (unexposed) in the last 90 days of life.**

|  | ONTARIO  (n=178,592) | | | NEW FOUNDLAND  (n=9,176) | | | BRITISH COLUMBIA  (n=58,556) | | | ALBERTA  (n=45,724) | | |
| --- | --- | --- | --- | --- | --- | --- | --- | --- | --- | --- | --- | --- |
| VARIABLE | **Received virtual care (Exposed)** | **Did not received virtual care**  **(Unexposed)** | **StdD** | **Received virtual care (Exposed)** | **Did not received virtual care**  **(Unexposed)*** | **StdD** | **Received virtual care (Exposed)** | **Did not received virtual care**  **(Unexposed)** | **StdD** | **Received virtual care (Exposed)** | **Did not received virtual care**  **(Unexposed)** | **StdD** |
| N (%) | 13,929  (7.8) | 164,663  (92.2) |  | 17  (0.2) | 9,159  (99.8) |  | 25,675 (43.8) | 32881  (56.2) |  | 22,227  (48.6) | 23,497  (51.4) |  |
|  |  |  |  |  |  |  |  |  |  |  |  |  |
| Age, Mean ± SD | 71.46 ± 15.38 | 74.76 ± 15.47 | 0.21 | 62.96 ± 17.18 | 75.84 ± 13.83 | 0.83 | 75.73 ± 13.56 | 71.23 ± 17.44 | 0.29 | 76.93 ± 14.03 | 68.04 ± 18.49 | 0.54 |
| Female Sex, n (%) | 6,320 (45.4) | 73,656 (44.7%) | 0.01 |  | 4281 (46.7) |  | 11,859 (46.2) | 12,976 (39.5) | 0.14 | 11,122 (50.0) | 9,234 (39.3) | 0.22 |
| Neighbourhood income quintile |  |  |  |  |  |  |  |  |  |  |  |  |
| 1 (Lowest) | 3,394 (24.4) | 41,972 (25.5) | 0.03 |  | 2240 (24.4) |  | 6,748 (26.3) | 9,934 (30.2) | 0.09 | 7,989 (35.9) | 8,659 (36.9) | 0.02 |
| 2 | 3,017 (21.7) | 36,446 (22.1) | 0.01 |  | 1932  (21.2) |  | 5,120 (19.9) | 7,231 (22.0) | 0.05 | 4,812 (21.7) | 5,216 (22.2) | 0.01 |
| 3 | 2,709 (19.4) | 31,519 (19.1) | 0.01 |  | 1939  (21.1) |  | 4,893 (19.1) | 6,011 (18.3) | 0.02 | 3,766 (16.9) | 3,836 (16.3) | 0.02 |
| 4 | 2,361 (17.0) | 27,509 (16.7) | 0.01 |  | 1842 (20.1) |  | 4,453 (17.3) | 5,058 (15.4) | 0.05 | 2,906 (13.1) | 2,913 (12.4) | 0.02 |
| 5 (Highest) | 2,405 (17.3) | 26,518 (16.1) | 0.03 |  | 1170  (12.7) |  | 4,360 (17.0) | 4,417 (13.4) | 0.10 | 2,642 (11.9) | 2,707 (11.5) | 0.01 |
| Missing | 43 (0.3) | 699 (0.4) | 0.02 |  | 53 (0.6) |  | 101 (0.4) | 230 (0.7) | 0.04 | 112 (0.5) | 166 (0.7) | 0.03 |
| Surname based Ethnicity |  |  |  |  |  |  |  |  |  |  |  |  |
| Chinese | 193 (1.4) | 4,203 (2.6) | 0.08 |  | 11 (0.1) |  | 1,187 (4.6) | 1,828 (5.6) | 0.04 | 421 (1.9) | 606 (2.6) | 0.05 |
| General | 13,543 (97.2) | 157,156 (95.4) | 0.1 |  | 9159 (100) |  | 24,006 (93.5) | 29,923 (91.0) | 0.09 | 21,587 (97.1) | 22,472 (95.6) | 0.08 |
| South-Asian | 193 (1.4) | 3,304 (2.0) | 0.05 |  | 13 (0.1) |  | 482 (1.9) | 1,130 (3.4) | 0.10 | 219 (1.0) | 419 (1.8) | 0.07 |
| Missing | 0 (0.0) | 0 (0.0) | 0 | -- | -- | -- | -- | -- | -- | 0 (0.0) | 0 (0.0) | 0.00 |
| Rural residence, n (%) |  |  |  |  |  |  |  |  |  |  |  |  |
| Yes | 3,439 (24.7) | 20,255 (12.3) | 0.32 |  | 5573  (60.7) |  | 4,112 (16.0) | 4,633 (14.1) | 0.05 | 5,113 (23.0) | 5,056 (21.5) | 0.04 |
| Missing | 42 (0.3) | 613 (0.4) | 0.01 |  | 44 (0.5) |  | 96 (0.4) | 219 (0.7) | 0.04 | 20 (0.1) | 72 (0.3) | 0.04 |
| Alcohol and substance use disorder | 727 (5.2) | 6,881 (4.2) | 0.05 |  | 258 (2.8) |  | 333 (1.3) | 1,237 (3.8) | 0.16 | 1,212 (5.5) | 2,217 (9.4) | 0.15 |
| Asthma | 2,158 (15.5) | 25,238 (15.3) | 0 |  | 912 (9.9) |  | 3,322 (12.9) | 4,328 (13.2) | 0.01 | 3,986 (17.9) | 4,084 (17.4) | 0.01 |
| Cancer | 5,589 (40.1) | 39,771 (24.2) | 0.35 |  | 2034 (22.1) |  | 9,258 (36.1) | 4,852 (14.8) | 0.50 | 7,512 (33.8) | 3,272 (13.9) | 0.48 |
| Cirrhosis | 348 (2.5) | 3,917 (2.4) | 0.01 |  | 170 (1.9) |  | 504 (2.0) | 598 (1.8) | 0.01 | 665 (3.0) | 557 (2.4) | 0.04 |
| COPD | 5,315 (38.2) | 58,247 (35.4) | 0.06 |  | 3042  (33.5) |  | 9,651 (37.6) | 10,733 (32.6) | 0.10 | 9,616 (43.3) | 8,381 (35.7) | 0.16 |
| Dementia | 1,304 (9.4) | 22,688 (13.8) | 0.14 |  | 1505 (16.4) |  | 2,193 (8.5) | 2,425 (7.4) | 0.04 | 4,625 (20.8) | 2,189 (9.3) | 0.33 |
| Diabetes | 4,831 (34.7) | 60,349 (36.7) | 0.04 |  | 4588 (50.0) |  | 8,733 (34.0) | 10,308 (31.3) | 0.06 | 7,954 (35.8) | 6,854 (29.2) | 0.14 |
| Heart failure | 3,324 (23.9) | 46,716 (28.4) | 0.1 |  | 2581 (28.1) |  | 8,601 (33.5) | 9,175 (27.9) | 0.12 | 7,896 (35.5) | 5,592 (23.8) | 0.26 |
| Hypertension | 9,394 (67.4) | 119,398 (72.5) | 0.11 |  | 7096 (77.4) |  | 18,083 (70.4) | 20,793 (63.2) | 0.15 | 17,249 (77.6) | 15,221 (64.8) | 0.29 |
| Non-psychotic disorder | 865 (6.2) | 9,225 (5.6) | 0.03 |  | 14 (0.15) |  | 334 (1.3) | 732 (2.2) | 0.07 | 1,390 (6.3) | 1,396 (5.9) | 0.01 |
| Psychotic disorder | 80 (0.6) | 1,242 (0.8) | 0.02 |  | 85 (0.93) |  | 133 (0.5) | 457 (1.4) | 0.09 | 183 (0.8) | 325 (1.4) | 0.05 |
| Renal failure | 2,713 (19.5) | 33,705 (20.5) | 0.02 |  | 1413  (15.3) |  | 5,650 (22.0) | 5,842 (17.8) | 0.11 | 5,167 (23.3) | 3,525 (15.0) | 0.21 |
| Stroke | 2,305 (16.5) | 31,875 (19.4) | 0.07 |  | 1791 (19.6) |  | 4,777 (18.6) | 5,329 (16.2) | 0.06 | 5,116 (23.0) | 3,485 (14.8) | 0.21 |
| Hospital frailty risk score (categorical) |  |  |  |  |  |  |  |  |  |  |  |  |
| 0. 0 | 2,001 (14.4) | 16,686 (10.1) | 0.13 |  | 2223  (24.2) |  | 4,301 (16.8) | 4,025 (12.2) | 0.13 | 2,659 (12.0) | 2,571 (10.9) | 0.03 |
| 1. 0.1 - 4.9 | 3,836 (27.5) | 37,396 (22.7) | 0.11 |  | 2441 (16.6) |  | 7,717 (30.1) | 8,271 (25.2) | 0.11 | 5,735 (25.8) | 5,015 (21.3) | 0.11 |
| 2. 5.0 - 8.9 | 1,943 (13.9) | 21,359 (13.0) | 0.03 |  | 1356 (0.14) |  | 3,734 (14.5) | 3,807 (11.6) | 0.09 | 3,421 (15.4) | 2,383 (10.1) | 0.16 |
| 3. 9.0 + | 2,916 (20.9) | 38,884 (23.6) | 0.06 |  | 1922 (21.0) |  | 4,529 (17.6) | 4,737 (14.4) | 0.09 | 6,868 (30.9) | 4,013 (17.1) | 0.33 |
| 4. No hospitalizations | 3,233 (23.2) | 50,338 (30.6) | 0.17 |  | 1234 (13.5) |  | 5,394 (21.0) | 12,041 (36.6) | 0.35 | 3,544 (15.9) | 9,515 (40.5) | 0.57 |
| Number of unique ED visits in past year^ⴕ^, Mean ± SD | 2.33 ± 4.09 | 1.55 ± 2.91 | 0.22 | 5.24 ± 3.77 | 2.66 ± 4.09 | 0.65 | 1.87 ± 2.90 | 1.35 ± 2.56 | 0.19 | 1.81 ± 3.51 | 1.25 ± 2.84 | 0.18 |
| Number of unique hospitalization episodes in past year, Mean ± SD | 1.12 ± 1.45 | 0.90 ± 1.36 | 0.16 | 1.88 ± 2.42 | 0.89 ± 1.44 | 0.5 | 1.15 ± 1.46 | 0.70 ± 1.27 | 0.33 | 1.10 ± 1.42 | 0.61 ± 1.19 | 0.37 |

*Smaller categories have been aggregated/suppressed to protect participant confidentiality

^ⴕ^Emergency Department visits that did not result in admission to hospital.

ED- Emergency Department; SD- Standard deviation; StdD- Standardized difference

**S1 Figure A: The Love plot illustrates the standardized differences in baseline characteristics across the four Canadian provinces among adults who died and either received (exposed) or did not receive (unexposed) virtual end-of-life care. Figure A shows the standardized differences during the COVID-19 pandemic. Red circles represent Ontario, Yellow triangles are for Newfoundland, Blue boxes for British Columbia, and Green diamonds for Alberta.**

**
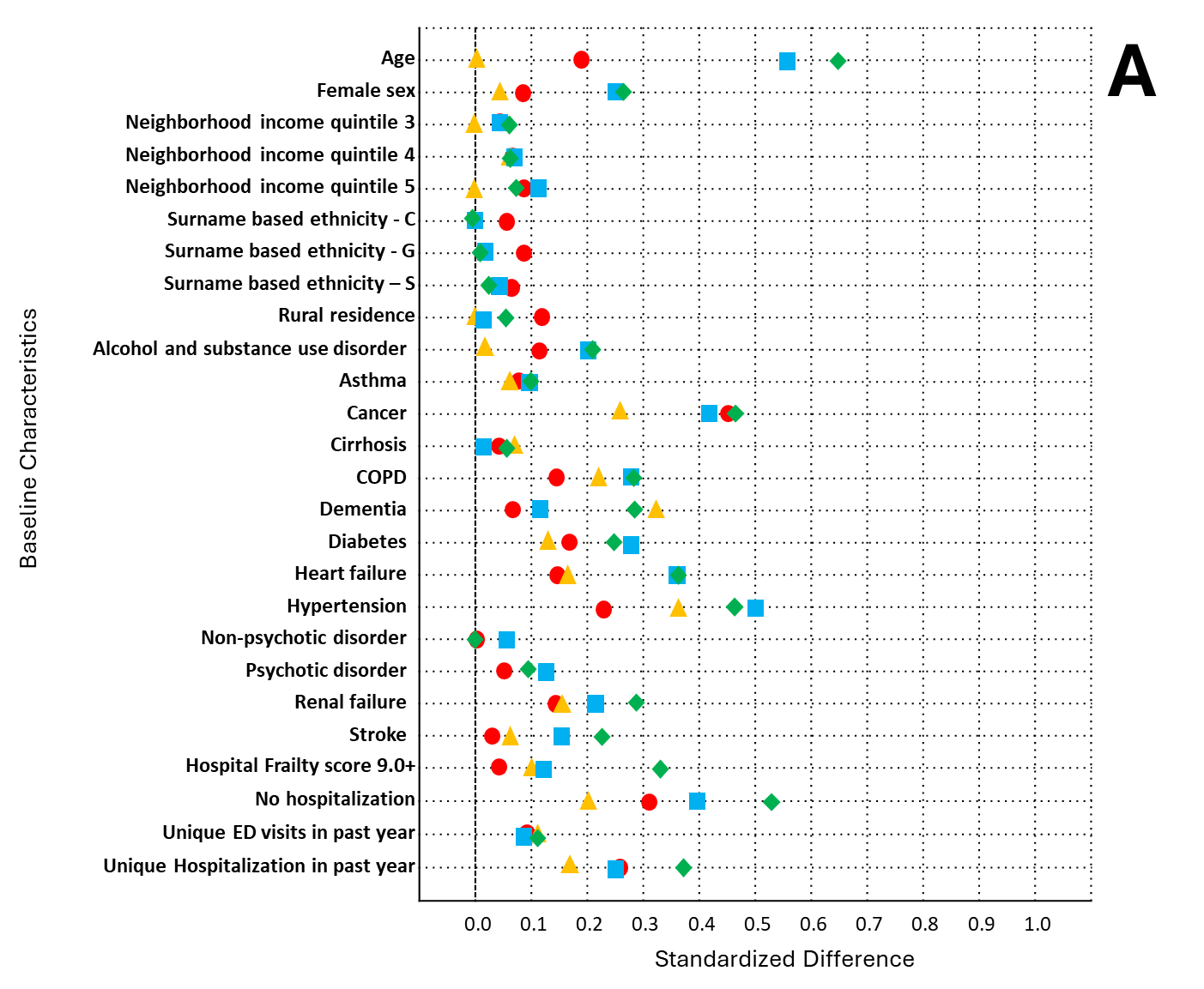
**

**S1 Figure B: The Love plot illustrates the standardized differences in baseline characteristics across the four Canadian provinces among adults who died and either received (exposed) or did not receive (unexposed) virtual end-of-life care. Figure B displays the differences in the pre-pandemic period. Red circles represent Ontario, Yellow triangles are for Newfoundland, Blue boxes for British Columbia, and Green diamonds for Alberta**

**
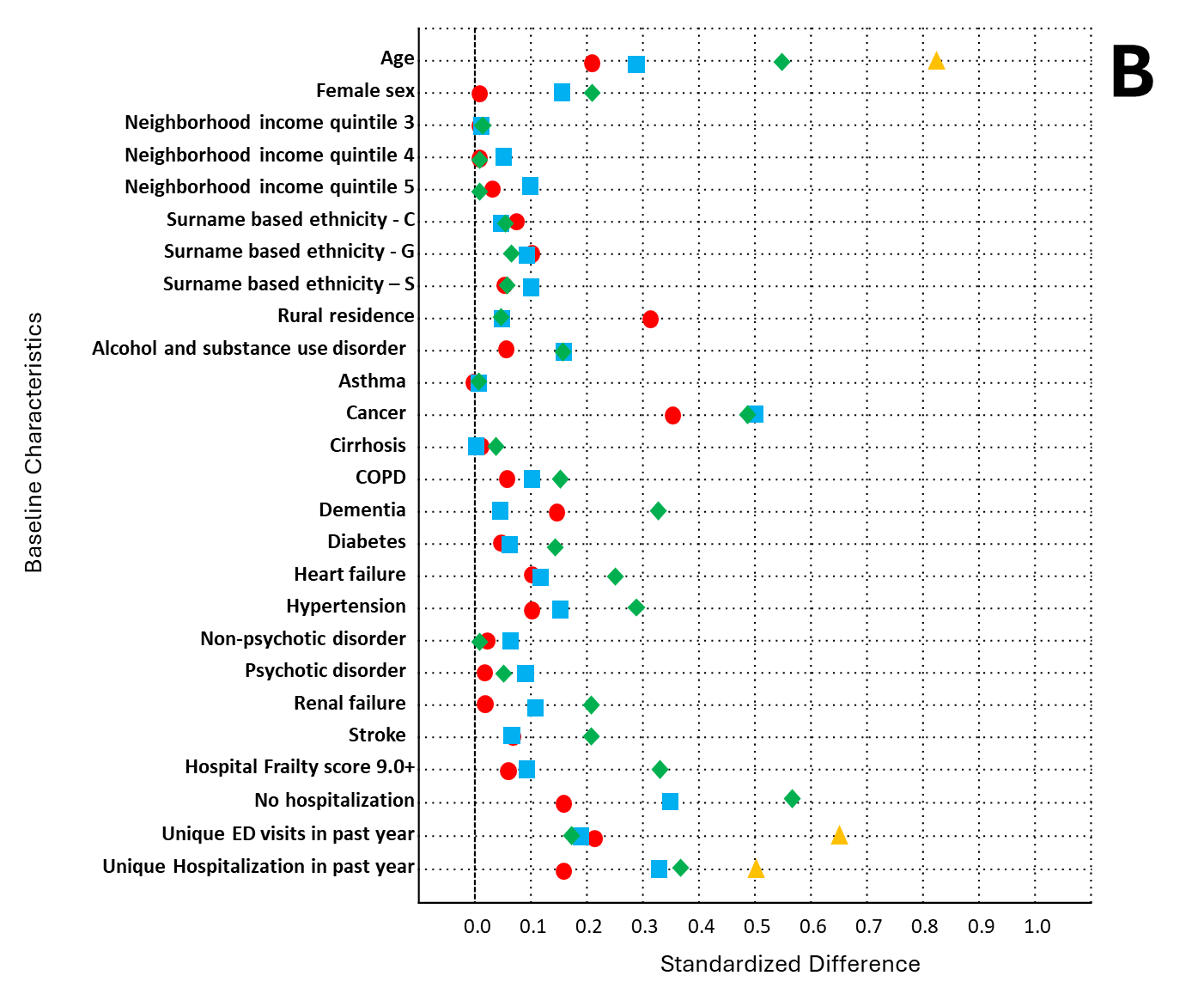
**
